# Association between *XRCC1* polymorphism 399 G->A and glioma among Caucasians: a systematic review and meta-analysis

**DOI:** 10.1186/1471-2350-13-97

**Published:** 2012-10-26

**Authors:** Daniel I Jacobs, Michael B Bracken

**Affiliations:** 1 Yale School of Public Health, New Haven, CT, USA; 2 Center for Perinatal, Pediatric and Environmental Epidemiology, Yale School of Public Health, New Haven, CT, USA

**Keywords:** Glioma, XRCC1, Polymorphisms, Meta-analysis

## Abstract

**Background:**

The x-ray cross complementing group 1 gene (*XRCC1*) is crucial to proper repair of DNA damage such as single-strand DNA breaks. A non-synonymous polymorphism in *XRCC1*, 399 G → A, has been shown to reduce effectiveness of such DNA repair and has been associated with the risk of certain cancers. The known risk for glioma from high dose ionizing radiation makes associations between this polymorphism and glioma of particular interest.

**Methods:**

A systematic literature review and meta-analysis was conducted to explore the association between *XRCC1* 399 G → A and glioma. Subgroup analyses by grade, gender, genotyping method, country in which study was conducted, and study size were conducted when data were available and validity of the results were assessed by influence analyses and exploration of potential publication bias.

**Results:**

Six studies were eligible for meta-analysis including data on 2,362 Caucasian glioma cases and 3,085 Caucasian controls. Pooled analysis yielded a significant association between the variant of interest and risk of glioma (OR = 1.17, 95% CI: 1.05-1.30) which was found to be disproportionately driven by a single study. Exclusion of this study, in an influence analysis, produced no statistically significant evidence of association with glioma (OR = 1.10, 95% CI: 0.98-1.23), and no evidence of publication bias.

**Conclusions:**

This meta-analysis does not suggest a major role of the *XRCC1* 399 G → A polymorphism in influencing risk of glioma among Caucasians. Future studies should report data separately for glioma subtypes to permit stratified analyses for Grade III and Grade IV glioma and examine other polymorphisms in this gene.

## Background

Malignant gliomas account for approximately 70% of adult malignant primary brain tumors in the United States and are associated with median survival of only 12 to 15 months among patients with glioblastoma, the most common type of glioma [1,2]. Research on the etiology of glioma over the past several decades has yielded few consistent findings; the only established environmental risk factor is exposure to therapeutic or high-dose ionizing radiation [3-5]. Studies of genetic susceptibility to glioma have been similarly inconsistent, although three recent genome-wide association studies (GWAS) have replicated findings at six susceptibility loci in *TERT, CCDC26, CDKN2A-CDKN2B, RTEL1, PHLDB1*, and *EGFR*[6-8]. Despite this progress, the majority of the risk of malignant gliomas remains unexplained.

Given the established link between ionizing radiation and glioma, it has been hypothesized that genetic variants of the DNA repair pathway may affect susceptibility to the disease. Specifically, x-ray cross complementing group 1 (*XRCC1*) is an important component of the base excision repair system, which is the predominant DNA repair pathway for small base lesions resulting from oxidation and alkylation damage [9]. The 33 kb *XRCC1* gene, located on chromosome 19q13.2-13.3, encodes a scaffolding protein which coordinates numerous protein-protein interactions, including with DNA ligase III and DNA polymerase at the site of damage [10,11].

Although over 300 single nucleotide polymorphisms (SNPs) have been identified in *XRCC1* (http://www.ncbi.nlm.nih.gov/projects/SNP), the most extensively studied has been rs25487 in codon 399 of exon 10, a non-synonymous G → A polymorphism changing arginine to glutamine. The frequency of the putative risk allele A has been shown to be 0.366, 0.274, and 0.111 in European American, east Asian, and African HapMap populations, respectively [12]. It has been suggested that this amino acid change results in deficient DNA repair, rendering carriers of the variant allele more susceptible to damage by environmental carcinogens [13,14]. The 399 G → A polymorphism has been shown to be associated with risk for numerous cancers including lung and prostate cancer among Asians, as well as breast cancer in both Asian and Caucasian populations [15-20]. As studies of the association of 399 G → A with the risk of glioma have been inconsistent, a systematic review and meta-analysis of such studies was conducted following PRISMA (Preferred Reporting Items for Systematic Reviews and Meta-Analyses) guidelines [21]. The aim was to elucidate the role of the polymorphism in glioma and to explore sources of heterogeneity among the identified studies.

## Methods

### Search strategy

A systematic literature search of the PubMed, EMBASE and Scopus databases was performed on October 25^th^, 2011 using search terminology [(XRCC1 OR x-ray cross complementing group 1) AND (glioma OR brain tumor)]. This was supplemented by a query of the HuGE Literature Finder based on a search of (glioma OR brain tumor) for gene *XRCC1*. Reference lists of identified studies were reviewed in order to identify additional relevant articles.

### Study selection

Included studies were required to meet all of the following conditions: 1) study employed a case–control design of human subjects; 2) outcome was microscopically-confirmed malignant glioma; and 3) data were presented on the genotype counts of cases and controls for the 399 G → A polymorphism in XRCC1. No language restriction was implemented, and corresponding authors were contacted in an attempt to obtain unreported genotype counts if studies were otherwise eligible. Genotype counts for cases and controls for the study by Liu et al. [22] were provided.

### Data extraction

The following data were extracted from each eligible article: last name of first author, date of publication, country in which study was performed, ethnicity of subjects, genotype counts of cases and controls, genotyping method and quality control, glioma subtype (grade III and/or grade IV), and age and gender composition of subjects. There was no blinding to the names of authors, journals, institutions, or funding sources.

### Statistical analysis

Deviation from Hardy-Weinberg equilibrium in control subjects was tested using a chi-squared goodness-of-fit test (*P* < 0.05 was considered significant). Assuming a fixed effects model, the inverse variance method was used to estimate separate genotype-specific summary odds ratios (OR) and 95% confidence intervals for the association between 399 G → A and risk of glioma. A fixed effects model was used due to the exhaustive nature of the literature review and a detailed exploration of possible sources of heterogeneity, as opposed to a random effects model which is not a solution for heterogeneity and assumes that identified studies are a subset of a larger pool of studies. Three pooled ORs were calculated: AA versus GG (OR_1_), AG versus GG (OR_2_), and AA versus AG (OR_3_). The resulting ORs were used to determine the most appropriate genetic model using a previously described approach [23]:

a) If OR_1_ = OR_3_ ≠ 1 and OR_2_ = 1, a recessive model is suggested

b) If OR_1_ = OR_2_ ≠ 1 and OR_3_ = 1, a dominant model is suggested

c) If OR_2_ = 1/OR_3_ ≠ 1 and OR_1_ = 1, an over dominant model is suggested

d) If OR_1_ > OR_2_ > 1 and OR_1_ > OR_3_ > 1 (or OR_1_ < OR_2_ < 1 and OR_1_ < OR_3_ < 1), a co-dominant model is suggested

After determination of the most appropriate genetic model, heterogeneity across studies was assessed using the *I*^*2*^ statistic [24]. To explore potential sources of heterogeneity, subgroup analyses were conducted according to gender, glioma subtype (Grade III vs. Grade IV), country (US vs. other), study size (<500 vs. ≥500), and genotyping method (PCR-RFLP vs. other). Influence analyses were also conducted, in which pooled estimates were calculated after omission of one study at a time to identify studies excessively influencing the summary estimate. Finally, publication bias was qualitatively assessed through use of a funnel plot of the log(standard error of the effect estimates) versus the effect estimates. Analyses were conducted using Review Manager Version 5.1.4 (Cochrane Collaboration, Oxford, UK) and R Version 2.13.1.

## Results

### Literature search and study selection

**Figure 1:**
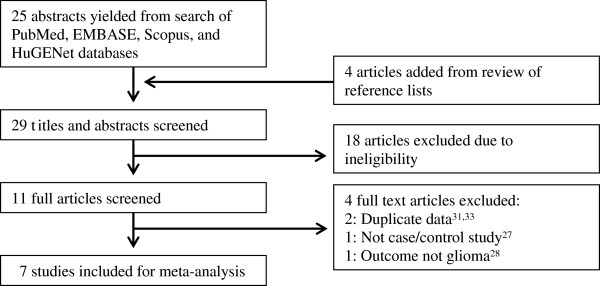
Flow diagram of systematic literature search.

The initial database search yielded 25 articles, and four additional articles were retrieved from a search of the reference lists of the originally identified articles. Screening of the titles and abstracts of these 29 articles for study eligibility led to the initial exclusion of 18 articles which did not meet eligibility requirements, leaving 11 full articles for review [22,25-34]. Of these articles, four were excluded due to not reporting on a case control study [28], providing duplicate data [31,33], and reporting the incorrect phenotype [29]. This systematic review resulted in the identification of seven eligible studies (Figure 1).

Of the seven studies retained for review, three were conducted in the United States [22,27,32], two in Turkey [25,34], one in Finland [30], and one in Brazil [26]. All studies reported on a mix of Grade III and Grade IV gliomas among exclusively Caucasian populations, based on DNA extracted from collected blood samples. Genotyping was conducted using restriction fragment length polymorphism PCR (PCR-RFLP) for all studies except Rajaraman et al. [32] which employed real-time TaqMan PCR, and Liu et al. [22] which used an array based approach. All control groups were in Hardy Weinberg equilibrium with the exception of that assembled by Custódio et al. [26] which deviated considerably (*P* < 0.001) and was consequently excluded from the meta-analysis. Thus, six studies [22,25,27,30,32,34] were included in the final meta-analysis with data on 2,362 unique cases and 3,085 controls (Table 1).

**Table 1:** Characteristics and genotype data extracted from eligible studies

**Study (Year)**		**Country**	**Ethnicity**	**Genotyping method**	**Glioma type**	**Mean age**		**Gender (% males)**	
Felini (2007)		USA	Caucasian	PCR-RFLP	Mixed	No data		No data	
Cengiz (2008)		Turkey	Caucasian	PCR-RFLP	Mixed	55.2		50.4	
Kiuru (2008)		Finland	Caucasian	PCR-RFLP	Mixed	No data		61.8	
Liu (2009)		USA	Caucasian	Array	Mixed	No data		56.8	
Rajaraman (2010)		USA	Caucasian	Taqman Real Time	Mixed	51.2		54.7	
Yosunkaya (2010)		Turkey	Caucasian	PCR-RFLP	Mixed	52.4		39.5	
	**Cases**				**Controls**				
**Study (Year)**	**N**	**GG**	**AG**	**AA**	**N**	**GG**	**AG**	**AA**	**HWE *p***
Felini (2007)	366	158	155	53	427	180	196	51	0.99
Cengiz (2008)	135	51	73	11	87	43	41	3	0.42
Kiuru (2008)	1,019	411	474	134	1,549	645	728	176	0.62
Liu (2009)	373	149	162	62	364	169	145	50	0.35
Rajaraman (2010)	350	142	164	44	478	205	201	72	0.40
Yosunkaya (2010)	119	15	67	37	180	91	71	18	0.87
Total	**2,362**	**926**	**1,095**	**341**	**3,085**	**1,333**	**1,382**	**370**	

### Association between *XRCC1* 399 G → A and Glioma

For the association between variants in 399 G → A and glioma, genotype-specific odds ratios OR_1_, OR_2_, and OR_3_ were 1.33 (95% CI 1.12-1.58, *P* = 0.001), 1.14 (95% CI 1.01-1.28, *P* = 0.03), and 1.16 (95% CI 0.98-1.37, *P* = 0.09), respectively. As these genotype-specific odds ratios are most suggestive of a dominant model, genotypes AG and AA were collapsed and the studies were meta-analyzed using a dominant model (AA + AG versus GG).

**Figure 2:**
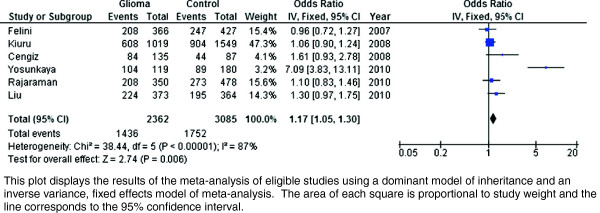
Forest plot of GG vs. AG+AA genotypes and association with glioma.

Results of the overall meta-analysis are presented in Figure 2. The pooled odds ratio among Caucasians for the association between 399 G → A AG + AA genotypes, as compared to GG genotype, and risk of glioma was statistically significant (OR = 1.17, 95% CI 1.05-1.30, *P* = 0.006), with indication of high among-study heterogeneity (*I*^*2*^ = 87%). Data were available to explore potential sources of this heterogeneity through stratification by gender, glioma subtype (Grade III vs. Grade IV), study country (US vs. other), genotyping method (PCR-RFLP vs. other), and study size (n < 500 vs. n ≥ 500), and results of these analyses are presented in Table 2. A statistically significant association was observed among females (OR = 2.27, 95% CI 1.45-3.57, *P* < 0.001), but this stratification was only feasible for one study [22] based on only 158 female cases. Furthermore, although significant associations were detected for studies with small sample size (OR = 3.09, 95% CI 2.06-4.65, *P* < 0.001), studies conducted outside of the US (OR = 1.22, 95% CI 1.05-1.42, *P* = 0.009), and studies employing PCR-RFLP genotyping (OR = 1.16, 95% CI 1.01-1.32, p = 0.03), it was clear that these associations were being driven by results of the study by Yosunkaya et al. [34]. An influence analysis demonstrated that there was no longer a statistically significant association between the *XRCC1* 399 G → A polymorphism and glioma after removing this study from the meta-analysis (OR = 1.10, 95% CI 0.98-1.23, *P* = 0.10), and there were no significant associations detected upon repeating previous subgroup analyses except for that among females as described above (Table 3). A funnel plot of the remaining studies did not indicate any evidence of publication bias (available upon request).

**Table 2:** **Results of overall and stratified analyses for the association of *XRCC1 *399 G → A and risk of glioma, all eligible studies**

**Stratification factor**	**No. of studies**	***I***^***2***^**(%)**	**Odds ratio**	**95% Confidence interval**	**P value**
Overall	6	87	1.17	1.05-1.30	0.006
Gender					
Male	1	NA	0.94	0.62-1.44	0.78
Female	1	NA	2.27	1.45-3.57	< 0.001
Glioma Subtype					
Grade III	2	0	1.05	0.89-1.25	0.56
Grade IV	2	46	1.20	0.97-1.47	0.09
Sample size					
< 500	2	92	3.09	2.06-4.65	< 0.001
≥ 500	4	0	1.08	0.96-1.21	0.19
Country					
US	3	8	1.11	0.94-1.31	0.22
Other	3	94	1.22	1.05-1.42	0.009
Genotyping method					
PCR-RFLP	4	92	1.16	1.01-1.32	0.03
Other	2	0	1.19	0.97-1.46	0.09

**Table 3:** **Results of overall and stratified analyses for the association of *XRCC1 *399 G → A and risk of glioma, Yosunkaya et al.**[34]**excluded**

**Stratification factor**	**No. of studies**	***I***^***2***^**(%)**	**Odds ratio**	**95% Confidence interval**	**P value**
Overall	5	7	1.10	0.98-1.23	0.10
Gender					
Male	1	NA	0.94	0.62-1.44	0.78
Female	1	NA	2.27	1.45-3.57	< 0.001
Glioma Subtype					
Grade III	2	0	1.05	0.89-1.25	0.56
Grade IV	2	46	1.20	0.97-1.47	0.09
Sample size					
< 500	1	NA	1.61	0.93-2.78	0.09
≥ 500	4	0	1.08	0.96-1.21	0.19
Country					
US	3	8	1.11	0.94-1.31	0.22
Other	2	53	1.09	0.94-1.27	0.26
Genotyping method					
PCR-RFLP	3	27	1.06	0.93-1.21	0.40
Other	2	0	1.19	0.97-1.46	0.09

## Discussion

Studies of the XRCC1 protein have demonstrated that it plays an important role in repairing single strand DNA breaks by participating in many processes including detecting such breaks and stabilizing proteins involved in the base excision repair system [35]. Because of the crucial role the protein plays, the effect of DNA variation in the coding sequence has been extensively explored in cancer and many other diseases. Studies have shown that the specific G → A substitution at codon 399 can induce conformational changes including the loss of α-helical structures that may be critical for effective protein-protein interactions [36], and that such variation corresponds with significantly reduced repair rates of irradiation-induced DNA damage [37].

Given the association of 399 G → A polymorphisms with other cancers [29] and the established risk of brain tumor development induced by ionizing radiation [3-5], these variants have been explored in numerous studies for association with glioma yet have yielded conflicting results. Despite the biologic plausibility of an association between *XRCC1* 399 G → A and risk of glioma, this meta-analysis does not provide evidence of such an association. The initial meta-analysis demonstrated a significant association of the polymorphism with glioma among Caucasians (OR = 1.17, 95% CI 1.05-1.30, *P* = 0.006) with high corresponding among-study heterogeneity (*I*^*2*^ = 87%). However, an influence analysis conducted by sequentially removing each study and recalculating the association and heterogeneity demonstrated that the study by Yosunkaya et al. [34], the smallest contributor to the meta-analysis (Figure 2), was disproportionately driving the apparent association and among-study heterogeneity. While the reported methodology of this study did not raise specific concerns, the observed odds ratio employing a dominant model of 7.09 (95% CI 3.83-13.11) is many times higher than has been observed for the association of this polymorphism and any cancer, and is almost certainly a statistical artifact of the small study sample. After exclusion of this study, there was not a statistically significant association demonstrated between the 399 G → A polymorphism and glioma (OR = 1.10, 95% CI 0.98-1.23, *P* = 0.10) among Caucasians, reduced heterogeneity (*I*^*2*^ = 7%), and there were no notable associations in stratified analyses.

While it is not possible to rule out the potential for bias in the identified studies, the five studies on which the final meta-analysis was based were deemed to be free of any major sources of bias which would compromise their validity. All studies were sufficiently clear in their description of recruitment methodology and participant characteristics, with the exception of reporting participant age in three studies [22,27,30]. All of the studies but one [25] discussed implementation of quality control methods during genotyping, such as use of control samples and replicates to assess concordance. However, only one study [27] reported blinding laboratory personnel to the case/control status of the samples. All studies reported matching controls to cases on age and gender, and all studies except one [25] reported matching on race/ethnicity or geographical location.

## Conclusions

Although there was no indication of systematic bias among the final eligible studies as described above, this meta-analysis may have been limited by the small sample sizes of some of the included studies, as well as the lack of data for some planned subgroup analyses. In particular, despite research indicating that the genetic landscape of Grade III versus Grade IV gliomas may differ considerably [38], only two of the included studies in this meta-analysis reported associations for the subtypes separately [22,30]. This limited our ability to explore associations with the *XRCC1* 399 G → A polymorphism that may be pertinent to one subtype but not the other. Future investigators should report genotype counts for glioma subtypes separately to permit stratified analyses. Despite these potential limitations, the current literature suggests no statistically significant association between the *XRCC1* 399 G → A polymorphism and glioma.

## Competing interests

The authors declare that they have no competing interests.

## Authors’ contributions

Both authors conceived and designed the study, performed the statistical analysis and interpretation, drafted the manuscript, revised for important intellectual content, and read and approved the final manuscript.

## Pre-publication history

The pre-publication history for this paper can be accessed here:

http://www.biomedcentral.com/1471-2350/13/97/prepub
